# WO_3_ Nanoparticles or Nanorods Incorporating Cs_2_CO_3_/PCBM Buffer Bilayer as Carriers Transporting Materials for Perovskite Solar Cells

**DOI:** 10.1186/s11671-016-1670-8

**Published:** 2016-10-18

**Authors:** Chih-Ming Chen, Zheng-Kun Lin, Wei-Jie Huang, Sheng-Hsiung Yang

**Affiliations:** Institute of Lighting and Energy Photonics, National Chiao Tung University, No. 301, Gaofa 3rd Road, Guiren District, Tainan, 71150 Taiwan, ROC

**Keywords:** Tungsten trioxide, Perovskite solar cells, Hydrothermal, Buffer bilayer

## Abstract

In this work, we demonstrate a novel carrier transporting combination made of tungsten trioxide (WO_3_) nanomaterials and Cs_2_CO_3_/PCBM buffer bilayer for the fabrication of perovskite solar cells (PSCs). Two different types of WO_3_, including nanoparticles and nanorods, were prepared by sol-gel process and hydrothermal method, respectively. Cs_2_CO_3_/PCBM buffer bilayer was inserted between WO_3_ and perovskite layers to improve charge transfer efficiency and formation of pinhole-free perovskite layer. Besides, the leakage current of the devices containing Cs_2_CO_3_/PCBM buffer bilayer was significantly suppressed. The optimized device based on WO_3_ nanoparticles and Cs_2_CO_3_/PCBM bilayer showed an open-circuit voltage of 0.84 V, a short-circuit current density of 20.40 mA/cm^2^, a fill factor of 0.61, and a power conversion efficiency of 10.49 %, which were significantly higher than those of PSCs without Cs_2_CO_3_/PCBM buffer bilayer. The results revealed that the combination of WO_3_ nanomaterials and Cs_2_CO_3_/PCBM bilayer provides an effective solution for improving performances of PSCs.

## Background

Perovskite solar cells (PSCs) have made impressive progress during several years due to high power conversion efficiency (PCE), low cost, and large-scale fabrication. The PCE values of PSCs have sharply risen from 3.8 to 21.1 % owing to excellent optical and electronic properties of organic/inorganic halide perovskite materials [1, 2], such as high absorption coefficient, long exciton diffusion length, and tunable band gap. The most used perovskite material is methylammonium lead iodide (CH_3_NH_3_PbI_3_ or MAPbI_3_) that is made of methylammonium iodide (MAI) and lead iodide (PbI_2_) [3]. Despite those advantages of PSCs, there still exist many challenges to commercialize PSC technologies in the near future, e.g., easy degradation from MAPbI_3_ to the starting material PbI_2_ under water and oxygen exposure, hysteresis under forward and reverse scans [4], and the usage of lead that is not friendly to our living environment.

The device architecture of PSCs is constructed with the configuration of fluorine-doped tin oxide (FTO)/electron transporting layer (ETL)/perovskite/hole transporting layer (HTL)/metal electrode. Many n-type metal oxide materials, including zinc oxide (ZnO), titanium dioxide (TiO_2_), tin dioxide_,_ and tungsten trioxide (WO_3_) [5–8], can be used as ETL in PSCs. On the other hand, p-type materials, such as vanadium oxide, nickel oxide, and molybdenum trioxide, can be utilized as HTL in PSCs [9–11]. Academic researches on TiO_2_ and ZnO-based ETLs for PSCs have been extensively reported in the literatures [12–15]. In fact, TiO_2_ exhibits relatively lower electrical conductivity among common inorganic electron transporting materials, implying lower transportation efficiency for carriers. High sintering temperature is also necessary for the preparation of high-quality TiO_2_ layers, which is unfavorable for low-cost manufacturing. The low-temperature process for the formation of TiO_2_ layer would limit its conductivity and electron transporting ability. On the other hand, ZnO possesses higher electron mobility and can be prepared via a simple fabrication process such as the hydrothermal method. However, there are still some drawbacks for ZnO to serve as ETL in PSCs. It is reported that ZnO could react with weak acids or bases [16], and its optoelectrical properties as well as surface morphology would be consequently affected. Furthermore, the perovskite may decompose to PbI_2_ upon thermal annealing on ZnO surface due to the reaction between released OH^−^ ions from ZnO and the component CH_3_NH_3_I. This usually results in the collapse of the perovskite structure and leads to poor device performance and stability of ZnO-based PSCs [17]. In addition to TiO_2_ and ZnO, WO_3_ is regarded as a potential candidate for the construction of PSCs. WO_3_ is an n-type material with electron mobility of 10–20 cm^2^/V s that is in the middle of TiO_2_ and ZnO [16, 18]. The optical band gap of WO_3_ ranges from 2.7 to 3.2 eV depending on different crystalline structures [19]. Furthermore, WO_3_ has high transmission in the visible region and excellent chemical stability which is better than that of ZnO [20]. After surveying previous literatures, we notice that electron transporting WO_3_ is less reported in the area of PSCs. In 2013, Amassian’s group firstly used untreated WO_3_ nanorods to fabricate PSCs with the highest PCE of 3.9 % [8]. The efficiency of PSC was promoted to 9.1 % by using TiCl_4_-treated WO_3_ nanorods as the ETL. In 2015, Ma et al. adopted the low-temperature amorphous WO_X_ as electron selective layer for PSCs with CH_3_NH_3_PbIXCl_3-X_ as the absorber [16]. The WO_X_ layer with a large quantity of nanocaves was used and the obtained PCE was 8.99 %. From a scientific and industrial viewpoint, developing alternatives or new types of materials is an essential issue to expand the diversity of research instead of adopting limited materials.

**Fig. 1:**
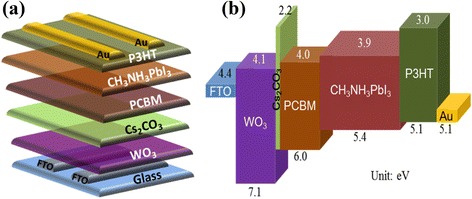
**a** Device structure and **b** energy level diagram of the PSCs

In this paper, we demonstrate the solution-processed WO_3_ nanoparticles layer (NL) and nanorod arrays (NAs) as the ETL for PSCs. To further improve the device performance of PSCs, ultra-thin cesium carbonate (Cs_2_CO_3_) and [6,6]-phenyl-C_60_ butyric acid methyl ester (PCBM) buffer layers were deposited on the above WO_3_ layers. Cs_2_CO_3_ is an inorganic compound that is commonly used as the electron injection material in organic light-emitting diodes and organic solar cells [21–23], or as a surface modification material for transparent conducting oxide [24]. PCBM is an electron acceptor that is usually blended with poly(3-hexylthiophene-2,5-diyl) (P3HT) or other low-band-gap conjugated polymers to fabricate bulk heterojunction solar cells [25, 26]. Based on the above discussion, we propose the new PSC structure with the configuration of FTO/WO_3_ NL or NAs/Cs_2_CO_3_/PCBM/MAPbI_3_/P3HT/Au which is illustrated in Fig. 1a. In this device architecture, P3HT acts as the HTL to collect holes from MAPbI_3_ layer, while WO_3_ covered with Cs_2_CO_3_/PCBM buffer bilayer is used to collect electrons. The energy level diagram of the whole device is illustrated in Fig. 1b. The lowest unoccupied molecular orbital of PCBM lies between the conduction band of WO_3_ and perovskite layers that is favored for electron extraction from the perovskite layer; the existence of PCBM layer can also prohibit carrier recombination at interfaces and back electron transfer from WO_3_ to perovskite layers. The ultra-thin Cs_2_CO_3_ layer is incorporated between WO_3_ and PCBM layers to increase electron transfer across neighboring layers. Electrons can transport from PCBM to WO_3_ through the Cs_2_CO_3_ layer under the tunneling effect due to its ultra-thin thickness [27]. Besides, this Cs_2_CO_3_ layer can also act as the blocking layer to prevent carrier recombination at the interface between WO_3_ and PCBM layers. On the other hand, hole transport from perovskite to P3HT is undisturbed, as shown in Fig. 1b. The optical, morphological, and crystallinity investigations of the two types of WO_3_ were carried out by miscellaneous techniques, including transmission spectroscopy, scanning electron microscopy (SEM), atomic force microscopy (AFM), and X-ray diffraction (XRD) analysis. The SEM and AFM images as well as photoluminescence (PL) quenching of the perovskite layer on pristine WO_3_ and WO_3_/Cs_2_CO_3_/PCBM layers were also performed to realize the role of Cs_2_CO_3_/PCBM bilayer on WO_3_ NL or NAs. Finally, WO_3_ NL or NAs-based PSCs without and with Cs_2_CO_3_/PCBM bilayers were fabricated and evaluated.

## Methods

### Characterization Methods

The UV-Vis absorption and transmission spectra of samples were measured by Princeton Instrument Acton 2150 spectrophotometer with a Xe lamp as a light source. The steady-state PL emission of the perovskite layer was measured using a He-Cd laser with double excitation wavelengths at 325/442 nm. The top-view and cross section SEM images of WO_3_ nanostructures and perovskite devices were obtained using JEOL 6700F FE-SEM. The surface morphology and roughness were obtained using a Bruker Innova AFM with tapping mode. The XRD patterns of samples were obtained using an X'Pert^3^ Powder X-ray Diffractometer, PANalytical Inc. with Cu-Kα radiation. The external quantum efficiency (EQE) measurement was conducted using a PV Measurements QEX10 instrument. The current density-voltage (*J*-*V*) curves of perovskite devices were performed using a Keithley 2400 digital sourcemeter under the sunlight simulator (Xenon Short Arc Lamp, USHIO UXL-10S) with irradiation intensity of 100 mW/cm^2^. The perovskite devices were masked with a metal aperture to define the active area of 4 mm^2^.

### Preparation of WO_3_ NL

A solution of tungsten acid (H_2_WO_4_, 0.375 g) was dissolved in 6 mL of 30 wt% hydrogen peroxide aqueous solution and heated at 140 °C. After the solution was concentrated from 6 to 1 mL, 9 mL of de-ionized water was added into the above solution and treated with ultra-sonication for 1 h, followed by filtration with 0.45 μm PTFE filter to form the precursor solution. The WO_3_ NL was spin-cast on the FTO substrate at 2000 rpm for 30 s and heated at 200 °C for 10 min. A second spin-coating step was carried out, and the film was sintered at 450 °C for 30 min to form the final WO_3_ NL with thickness of about 50 nm, as verified by AFM step height measurement.

### Preparation of WO_3_ NAs

WO_3_ NAs were prepared via the hydrothermal method. Sodium tungstate dihydrate (Na_2_WO_4_·2H_2_O, 4.125 g) was dissolved in 12.5 mL of de-ionized water and stirred at room temperature for 1 h. Into the above solution, 0.2 M hydrochloric acid aqueous solution was then added dropwise until the pH value of the solution was adjusted from 9.2 to 2.4. Afterward, de-ionized water was added till the total volume of the solution is 125 mL, followed by ultrasonic treatment for 30 min to form the precursor solution. A bath solution containing 20 mL of the precursor solution and 0.3 g of sodium chloride was poured into a teflon-lined autoclave. The WO_3_ NL on the FTO substrate was immersed with the surface downwards in the bath solution, and the autoclave was placed in a preheated oven at 170 °C for 120 min. The substrates were then taken out and cleaned with de-ionized water, acetone, and isopropyl alcohol (IPA) sequentially, followed by calcination at 500 °C for 1 h. The height of the obtained WO_3_ NAs is 300 nm, as verified by a SEM cross section.

### Fabrication of Perovskite Solar Cells

The FTO conductive substrates were sequentially cleaned with detergent, de-ionized water, acetone, and IPA with ultra-sonication for 30 min, dried at 100 °C for 10 min to remove the solvent residue, and followed by UV-ozone treatment for 20 min. WO_3_ NL or NAs were prepared on the pre-cleaned FTO substrates according to the processes in the previous section. A solution of Cs_2_CO_3_ in de-ionized water (2 mg/mL) was dropped on the WO_3_ layer (NL or NAs); after waiting for 30 s, the substrate was spin-coated at 2500 rpm for 60 s, followed by heating treatment at 300 °C for 30 min in ambient environment. After cooling to room temperature, a solution of PCBM in chlorobenzene (20 mg/mL) was dropped on the Cs_2_CO_3_ layer; after waiting for 5 s, the substrate was spin-coated at 3000 rpm for 30 s, followed by heating treatment at 120 °C for 10 min in a vacuum oven. The perovskite layer CH_3_NH_3_PbI_3_ was formed via the two-step sequential deposition procedure. First, a PbI_2_ solution in DMF (462 mg/mL) was spin-coated on the WO_3_ layer at 5000 rpm for 30 s, and the substrate was placed in a petridish for 10 min, followed by heating treatment at 100 °C for 20 min in a vacuum oven. Second, a MAI solution in IPA (40 mg/mL) was dropped on the PbI_2_ layer; after waiting for 60 s, it was then spun at 5000 rpm for 30 s. The substrate was then heated at 100 °C for 20 min in a vacuum oven. The P3HT layer was formed by spin-coating from its solution in chlorobenzene (20 mg/mL) and heated at 100 °C for 30 min. Finally, gold electrodes were thermal-evaporated on the P3HT layer under a base pressure ~10^−6^ torr.

## Results and Discussion

### Nanostructural, Electrical, and Optical Analyses of Materials

**Fig. 2:**
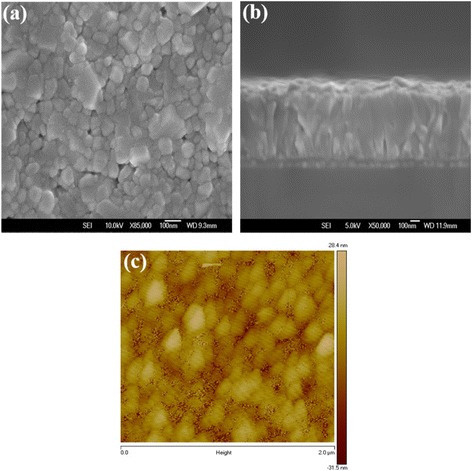
**a** Top-view, **b** cross section SEM, and **c** AFM topographic images of WO_3_ NL

**Fig. 3:**
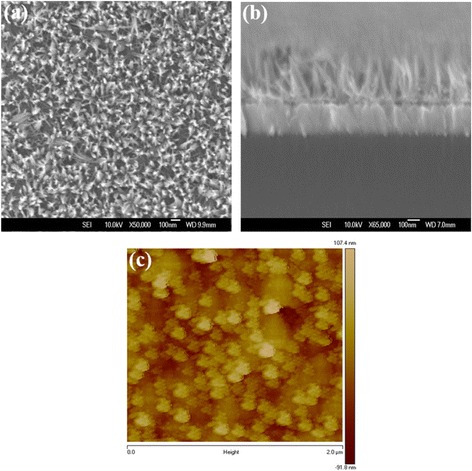
**a** Top-view, **b** cross section SEM, and **c** AFM topographic images of WO_3_ NAs

**Fig. 4:**
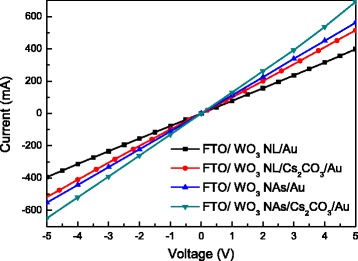
Linear sweep voltammetry curves of the four electron-only devices

**Fig. 5:**
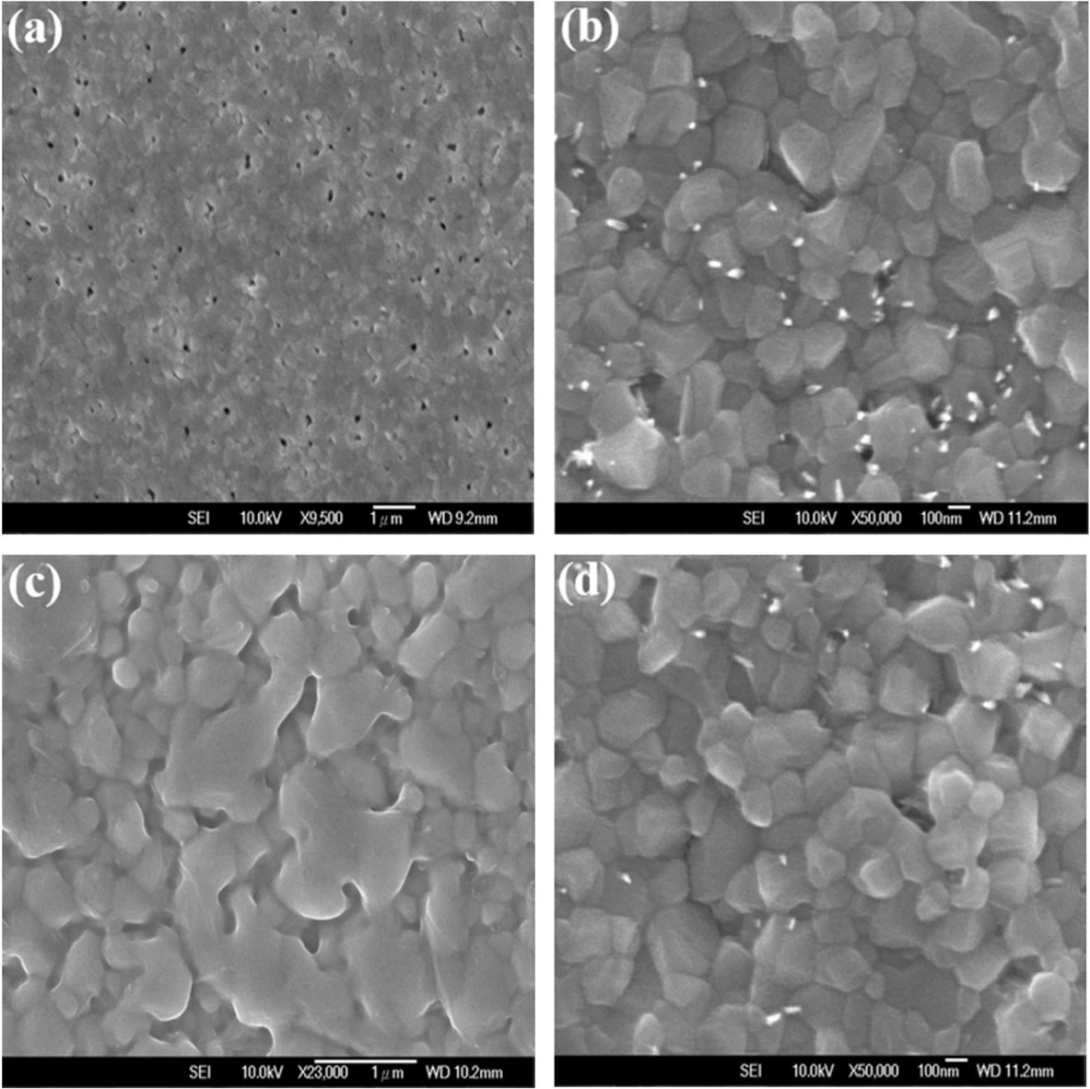
Top-view SEM images of the perovskite layers deposited **a** WO_3_ NL, **b** WO_3_ NAs, **c** WO_3_ NL/Cs_2_CO_3_/PCBM, and **d** WO_3_ NAs/Cs_2_CO_3_/PCBM

**Fig. 6:**
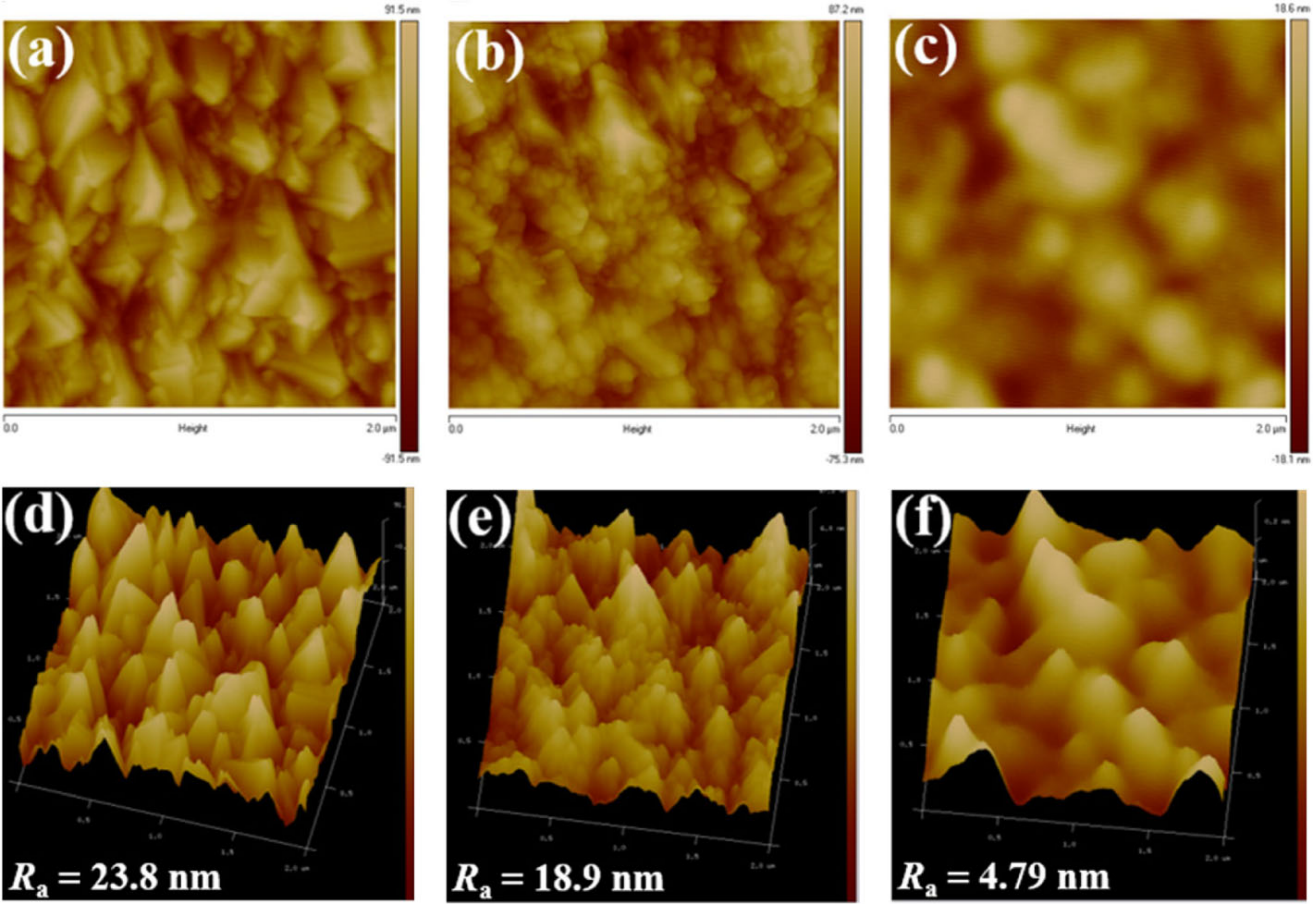
AFM topographic and 3D images of **a**, **d** bare FTO, **b**, **e** FTO/WO_3_ NL, and **c**, **f** FTO/WO_3_ NL/Cs_2_CO_3_/PCBM. The corresponding *R*_a_ values of different films are also included

Figure 2a, b show the top-view and cross section SEM images of WO_3_ NL, respectively. It is clearly seen that most WO_3_ nanoparticles are tightly formed with diameters ranging from 50 to 100 nm, while the thickness of WO_3_ NL is estimated to be 80–100 nm. Compared to some flat layers, this kind of WO_3_ NLs provides a larger surface area that is beneficial for carrier extraction and photovoltaic performance [28]. Some pinholes and aggregates are also observed on the surface, which can be further reduced by covering with adequate buffer layers such as Cs_2_CO_3_/PCBM bilayer to prevent trapping of electrons and direct contact of FTO and perovskite layer. Figure 2c shows the topographic image of WO_3_ NL deposited on the quartz substrate by AFM. The particle size and surface morphology of WO_3_ nanoparticles are consistent with those observed from SEM images. The surface roughness (*R*_a_) is calculated to be about 5.0 nm, indicative of a smooth surface of WO_3_ NL. The top-view and cross section SEM images of WO_3_ NAs are shown in Fig. 3c, b, respectively. Well-aligned WO_3_ NAs with length of about 300 nm are vertically grown on the WO_3_ NL. The total surface areas of WO_3_ NAs are supposed to be larger than the NL due to the high aspect ratio of nanorods, which might show a positive effect on carrier extraction and transportation. The surface morphology of WO_3_ NAs investigated by AFM technique is shown in Fig. 3c. Rod-like shapes and a higher *R*_a_ value of 32 nm are observed for the prepared WO_3_ NAs in this study. To further compare the conductivity of WO_3_ NL and NAs, simple electron-only devices with the configuration of FTO/WO_3_/Au and FTO/WO_3_/Cs_2_CO_3_/Au were fabricated; the current-voltage characteristics of four devices were measured and shown in Fig. 4. It is seen that WO_3_ NAs show higher electrical conductivity and lower resistance compared to the WO_3_ NL. The results suggest that NAs-type of WO_3_ is more favorable for electron transport than the NL one. Furthermore, the electron current of the device FTO/WO_3_/Cs_2_CO_3_/Au is even higher than that without the Cs_2_CO_3_ layer, indicating an enhanced electron-extraction ability of this ETL system. According to the previous literatures [23, 29], a thin layer of Cs_2_CO_3_ is capable of lowering the work function of the underlying layer. In this study, the thin Cs_2_CO_3_ layer lowers the work function of the WO_3_ layer and thereby increases electron-extraction ability in this combined WO_3_/Cs_2_CO_3_ system. Figure 5a, b show the top-view SEM images of perovskite films deposited on the WO_3_ NL and NAs, respectively. There are many pinholes to be observed on the perovskite films, indicating higher probability of electron trapping and less light absorption in both cases. A similar observation has been reported in the previous literature [16], stating hole-forming and poor coverage of perovskite on the WO_3_ layer. To solve this problem, a thin Cs_2_CO_3_/PCBM bilayer is proposed to be inserted between WO_3_ and perovskite layers in this study. After depositing the Cs_2_CO_3_/PCBM bilayer, the *R*_a_ values of WO_3_ NL and NAs were reduced to 0.874 and 30 nm, respectively, which is beneficial for the deposition of perovskite layer. From Fig. 5c, d, a pinhole-free perovskite surface with larger grain size was obtained on both WO_3_ layers covered with a Cs_2_CO_3_/PCBM bilayer, revealing higher surface coverage of the perovskite layer. Till now several techniques have been proposed to form a pinhole-free perovskite layer, such as solvent annealing [30], moisture-control method [31], inverted thermal annealing [32], addition of methylammonium bromide precursor [33], and the selection of lead sources [34]. Here we demonstrate an alternative way to reduce pinholes on perovskite layer by the incorporation of a Cs_2_CO_3_/PCBM bilayer. Besides, we notice that the perovskite with a larger grain size of 250–650 nm was formed on WO_3_ NL; on the contrary, the perovskite with smaller crystallites of 200–400 nm was found on WO_3_ NAs. It is concluded that the formation of perovskite layer is strongly influenced by the type of WO_3_ layer. Furthermore, some WO_3_ nanorods are observed to penetrate through the perovskite layer to the top surface, as shown in Fig. 5b. This would lead to direct contact of WO_3_ NAs and hole transporting P3HT layer and result in leakage current to lower photovoltaic properties of devices. The phenomenon of nanorod penetration can be largely suppressed by the incorporation of a Cs_2_CO_3_/PCBM bilayer on WO_3_ NAs, as revealed in Fig. 5d. The penetration of WO_3_ nanorods may be further suppressed by increasing the thickness of the perovskite layer; however, the carrier transport and diffusion length would also be affected that is unfavorable for device performance. In order to comprehend morphologies and roughness change for the deposition of Cs_2_CO_3_/PCBM bilayer on WO_3_ NL, the AFM images and *R*_a_ values of the bare FTO, FTO/WO_3_ NL, and FTO/WO_3_ NL/Cs_2_CO_3_/PCBM were investigated and shown in Fig. 6. The polycrystalline feature of FTO surface in Fig. 6a, d is clearly changed to nanoparticle-aggregated morphology after depositing WO_3_ NL on top of FTO shown in Fig. 6b, e, and corresponding *R*_a_ value is decreased from 23.8 to 18.9 nm, indicating that FTO surface is covered and smoothed by WO_3_ NL. Moreover, amorphous surface morphology and eliminated grain boundary are obtained for FTO/WO_3_ NL/Cs_2_CO_3_/PCBM with the smallest *R*_a_ value of 4.79 nm, as shown in Fig. 6c, f. In other words, the surface properties of WO_3_ NL are further modified by the coverage of Cs_2_CO_3_/PCBM bilayer. The very smooth top surface is particularly suitable for the deposition of highly dense and pinhole-free perovskite layers.

**Fig. 7:**
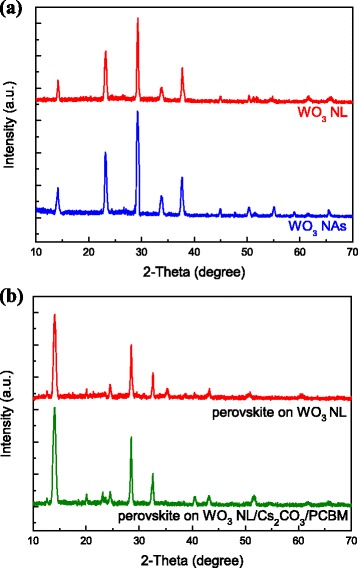
XRD patterns of **a** WO_3_ NL and NAs. **b** perovskite deposited on WO_3_ NL without and with Cs_2_CO_3_/PCBM bilayer

The XRD patterns of WO_3_ NL and NAs are shown in Fig. 7a. All diffraction peaks are well indexed to the standard diffraction pattern of hexagonal phase of WO_3_ (JCPDS 00-033-1387). Besides, these diffraction peaks are sharp and strong, indicating high-degree crystallization of the prepared WO_3_ samples in this research. The two main diffraction peaks located at *2θ* = 23.4° and 29.3° are assigned to (001) and (200) planes, respectively, which is in good accordance with the previous report [35]. The XRD result suggests that the WO_3_ NAs are grown along (001) direction and aligned with the *c*-axis [36], not parallel to the FTO substrate. The crystalline phases of WO_3_ NL and NAs are concluded to be similar because of the same *2θ* position of diffraction peaks in Fig. 7a. Furthermore, the intensities of diffraction peaks of WO_3_ NAs is stronger than those of WO_3_ NL, revealing a higher degree of crystallinty of WO_3_ NAs. Figure 7b shows the XRD patterns of perovskite film deposited on WO_3_ NL without and with a Cs_2_CO_3_/PCBM bilayer. First, three main diffraction peaks of perovskite film at *2θ* = 14.1°, 28.5°, and 32.6° are assigned to (110), (220), and (310) planes, respectively, which are identical to the previous literature [37]. The rest of the weaker diffraction peaks located at *2θ* = 24.5°, 40.5°, and 43.1° are assigned to (202), (224), and (314) planes, respectively. Second, those *2θ* positions of diffraction peaks were unchanged after the incorporation of Cs_2_CO_3_/PCBM bilayer, indicating that the crystalline phase of perovskite was preserved. Third, the intensities of XRD patterns of perovskite on WO_3_ NL/Cs_2_CO_3_/PCBM are increased compared to those on untreated WO_3_ NL, revealing that the degree of crystallinity of perovskite is enhanced. The increased diffraction intensity can be realized by better crystallization of perovskite on a flatter WO_3_ NL/Cs_2_CO_3_/PCBM surface, which has been identified by AFM observation. The strengthened crystallization of perovskite can also be verified by SEM images in Fig. 6, exhibiting much larger perovskite crystals on Cs_2_CO_3_/PCBM-modified WO_3_ layer. This is the first report on the improvement of perovskite crystallization by incorporating Cs_2_CO_3_/PCBM bilayer on metal oxide layers. Besides, a small diffraction peak located at *2θ* = 12.6° was found and assigned to the starting material PbI_2_, implying that the precursors were not compeletely transformed to perovskite structure. The existence of residual PbI_2_ is generally thought to be harmful to device performance, since carriers might be trapped and light absorption could be affected [38]. On the contrary, some research groups declare that the presence of a small amount of PbI_2_ can slightly enhance the device performance and suppress the carrier recombination [39–41]. It seems that the effect of residual PbI_2_ in PSCs is sometimes controversial.

**Fig. 8:**
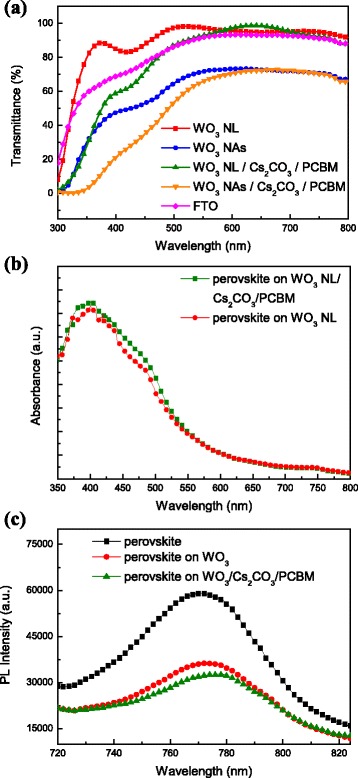
**a** Transmission spectra of different WO_3_ without and with Cs_2_CO_3_/PCBM bilayer. **b** Absorption spectra of the perovskite on WO_3_ NL without and with Cs_2_CO_3_/PCBM bilayer. **c** PL spectra of the perovskite on FTO, WO_3_ NL, and WO_3_ NL/Cs_2_CO_3_/PCBM

The transmission spectra of WO_3_ layer (NL and NAs) without and with Cs_2_CO_3_/PCBM bilayer are shown in Fig. 8a. The WO_3_ NL possesses the highest transmittance up to 80–95 % that is higher than WO_3_ NAs and FTO substrate in the visible region. This is beneficial for incident photons to enter devices and to be absorbed by the active layer. After the insertion of Cs_2_CO_3_/PCBM bilayer, the transmittance is decreased for both WO_3_ NL and NAs, which is due to the absorption nature of PCBM in the region from 300 to 500 nm. The absorption spectra of the perovskite layer on WO_3_ NL without and with Cs_2_CO_3_/PCBM bilayer are shown in Fig. 8b. The main absorption of the perovskite on WO_3_ NL with Cs_2_CO_3_/PCBM bilayer grows stronger in the range of 350 and 550 nm compared with that on the untreated WO_3_ NL. This is because a highly dense and pinhole-free perovskite layer is formed on Cs_2_CO_3_/PCBM-modified WO_3_ NL, which can absorb more incident light. More excitons are expected to generate inside the perovskite layer under sunlight exposure, and higher device efficiency would be achieved. It is also noted that the absorption edge of the perovskite is found around 770 nm that is similar on both untreated and Cs_2_CO_3_/PCBM-modified WO_3_ NLs. The steady-state PL spectra of the perovskite layer on the bare FTO substrate, WO_3_ NL, and WO_3_ NL/Cs_2_CO_3_/PCBM are shown in Fig. 8c. The max PL emission of the perovskite is located at 770 nm under excitation of a He-Cd laser source, which is in accordance with the previous report [42]. Distinct PL quenching of the perovskite is found when depositing on WO_3_ NL, implying better charge carrier extraction across the interface between the perovskite and WO_3_ NL [43]. By depositing Cs_2_CO_3_/PCBM bilayer on WO_3_ NL, the PL emission of the perovskite is further prohibited, indicating more effective carrier extraction and reduced recombination. In other words, the incorporation of Cs_2_CO_3_/PCBM bilayer facilitates carrier transfer from the perovskite layer to WO_3_ NL, which will help to improve short-circuit current density (*J*_SC_) and performance of PSCs. Similar behaviors including enhanced absorption and PL quenching of the perovskite layer can also be observed when incorporating Cs_2_CO_3_/PCBM bilayer on WO_3_ NAs.

### Photovoltaic Properties of Perovskite Devices

To evaluate photovoltaic performance of PSCs, five different device configurations were designed and are listed as follows:

- Device A: FTO/WO_3_ NL/MAPbI_3_/P3HT/Au
- Device B: FTO/WO_3_ NL/Cs_2_CO_3_/PCBM/MAPbI_3_/P3HT/Au
- Device C: FTO/WO_3_ NAs/MAPbI_3_/P3HT/Au
- Device D: FTO/WO_3_ NAs/Cs_2_CO_3_/PCBM/MAPbI_3_/P3HT/Au
- Device E: FTO/MAPbI_3_/P3HT/Au

**Fig. 9:**
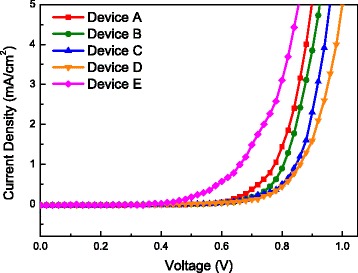
*J*-*V* curves of devices A through E in the dark

The *J*-*V* curves of all PSCs measured in the dark are shown in Fig. 9. The dark currents arose at 0.6 and 0.75 V for the devices based on untreated WO_3_ NAs and WO_3_ NL, respectively. After the insertion of Cs_2_CO_3_/PCBM bilayer, the dark currents arose at 0.7 and 0.85 V for the devices based on WO_3_ NAs and WO_3_ NL, respectively. The retardation in dark current brought by Cs_2_CO_3_/PCBM bilayer can be observed for both WO_3_ NL and NAs-based PSCs; moreover, the dark current of the device based on WO_3_ NL was more prohibited than that based on WO_3_ NAs. This could be due to the penetration of WO_3_ nanorods through the perovskite layer which was confirmed by SEM observation in Fig. 5b, d. The retardation in dark current means less leakage current and prohibition of back flow of electrons from WO_3_ to perovskite layer [44]. The suppression of leakage current can be realized by the passivation effect, i.e., the surface defects of WO_3_ layer were passivated by the incorporation of Cs_2_CO_3_/PCBM bilayer. Besides, a better energy level alignment was achieved by the insertion of Cs_2_CO_3_/PCBM bilayer shown in Fig. 1b, indicative of forming an ohmic contact from the FTO to perovskite layer for electron transport. Fewer electrons can be trapped and recombination rate is consequently decreased, leading to better device performance such as *J*_SC_ and fill factor (FF). By comparison, the dark current of the reference device E without WO_3_ ETL arose around 0.4 V, implying the highest recombination rate of carriers among all devices.

**Fig. 10:**
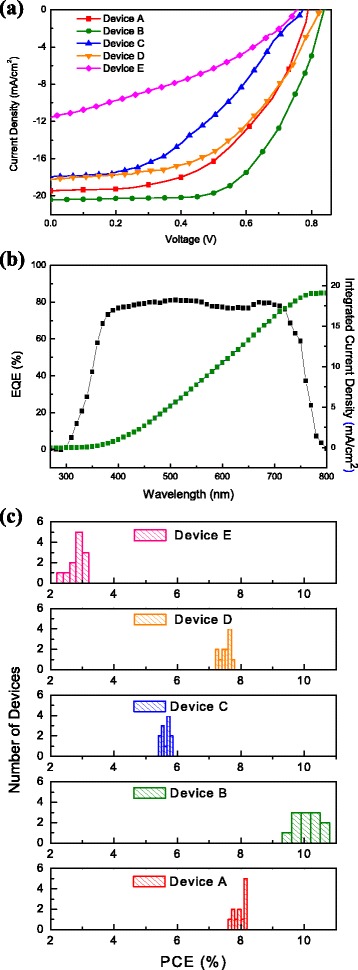
**a** *J*-*V* curves of devices without and with Cs_2_CO_3_/PCBM bilayer. **b** EQE spectrum and integrated current density of the best device B. **c** The histogram of the PCE values of 12 devices for devices A through E

The *J*-*V* curves of the five devices A, B, C, D, and E under 100 mW/cm^2^ of sunlight irradiation are depicted in Fig. 10a, and the photovoltaic properties of all devices are summarized in Table 1. Device A showed an open-circuit voltage (*V*_OC_), *J*_*S*C_, FF, and PCE values of 0.78 V, 19.45 mA/cm^2^, 0.53, and 8.13 %, respectively, which were much higher than the previous report based on the same device configuration and perovskite material [8]. By introducing Cs_2_CO_3_/PCBM bilayer, the device B reached the highest PCE of 10.49 % that can be attributed to the improvement of *J*_SC_ (20.40 mA/cm^2^) and FF (0.61) compared with device A. The *J*_SC_ improvement can be realized from several experimental observations, including increased absorption in the visible range, enlarged grain size of perovskite crystals from SEM and AFM observation, and suppression of carrier recombination from PL quenching and dark current measurements. The explanations to the FF improvement include pinhole-free surface, reduced roughness of WO_3_ layer, prohibited leakage current, and lower recombination rate. The performance of the reference device E without WO_3_ was also evaluated and listed in Table 1. The poor device performance of device E indicates the necessity of WO_3_ ETL in the proposed PSC structure. To verify conversion efficiency versus wavelength and integrated photocurrent under light irradiation, the best device B was taken as an example and investigated. The EQE spectrum and integrated current density of device B are shown in Fig. 10b, revealing a quite stable EQE around 80 % from 400 to 700 nm. An integrated current density of 19.1 mA/cm^2^ is obtained, which is in good agreement with measured *J*_SC_ under the sunlight simulator. Turning to devices C and D based on WO_3_ NAs, the device performance was also enhanced by the insertion of Cs_2_CO_3_/PCBM bilayer. Besides, we notice that the *J*_SC_ and PCE of the devices based on WO_3_ NAs are not as high as those using WO_3_ NL as ETL, though WO_3_ NAs are characterized to own higher conductivity. The reasons to this phenomenon could be due to poorer surface coverage of perovskite layer on WO_3_ NAs and 20 % lower transmittance of WO_3_ NAs in the visible region; less incident photons would enter devices and be absorbed by perovskite layer, resulting in lower charge collection and *J*_SC_. Moreover, direct contact between ETL (WO_3_ NAs) and HTL (P3HT layer) from penetration of WO_3_ nanorods through perovskite layer leads to higher leakage current and lower FF. To examine the reproducibility of WO_3_-based PSCs without and with Cs_2_CO_3_/PCBM bilayer, PCE of 12 individual devices for devices A through E were measured and collected, and the statistical distributions of those devices are depicted in Fig. 10c. The averaged PCE values of devices A, B, C, D, and E are 7.96 ± 0.17, 10.10 ± 0.39, 5.63 ± 0.16, 7.50 ± 0.21, 2.80 ± 0.37, respectively, which are also listed in Table 1. It is clearly seen that a high level of reproducibility and reliability of PCE for each kind of device is obtained, and effective improvement in PCE can be deduced with the incorporation of Cs_2_CO_3_/PCBM bilayer on WO_3_-based PSCs.

**Table 1:** Photovoltaic properties of the four PSCs with different configurations

Devices	*V*_OC_ (V)	*J*_SC_ (mA/cm^2^)	FF	PCE (%)	Avg. PCE (%)^a^
A	0.78	19.45	0.53	8.13	7.96 ± 0.17
B	0.84	20.40	0.61	10.49	10.10 ± 0.39
C	0.78	17.90	0.41	5.79	5.63 ± 0.16
D	0.82	18.20	0.52	7.71	7.50 ± 0.21
E	0.76	11.54	0.36	3.17	2.80 ± 0.37

^a^Average PCE data are obtained from 12 devices

## Conclusions

We demonstrated the insertion of Cs_2_CO_3_/PCBM bilayer between WO_3_ and perovskite layers to enhance the performance of PSCs. By inserting Cs_2_CO_3_/PCBM bilayer, the morphologies and roughness of WO_3_ were modified for better deposition of highly dense and pinhole-free perovskite layers. The degree of crystallinity of the perovskite as well as its absorption from 350 to 550 nm was also increased due to the incorporation of a Cs_2_CO_3_/PCBM bilayer. Besides, the prohibited PL emission of the perovskite brought by the Cs_2_CO_3_/PCBM bilayer indicates more effective carrier extraction and reduced recombination, which facilitates the carrier transfer from perovskite to WO_3_ layers. An optimized PCE of 10.49 % and a high *J*_SC_ value of 20.40 mA/cm^2^ were achieved with the device based on WO_3_ NL covered with Cs_2_CO_3_/PCBM bilayer. Lower PCE of 7.71 % and *J*_SC_ of 18.2 mA/cm^2^ of the device based on WO_3_ NAs were obtained due to the penetration of WO_3_ nanorods through the perovskite layer, larger surface roughness, and lower transmission of WO_3_ NAs compared with WO_3_ NL.

## Abbreviations

AFM: Atomic force microscopy
EQE: External quantum efficiency
ETL: Electron transporting layer
FF: Fill factor
HTL: Hole transporting layer
*J*_SC_: Short-circuit current density
J-V: Current density-voltage
MAI: Methylammonium iodide
MAPbI_3_: Methylammonium lead iodide
NAs: Nanorod arrays
NL: Nanoparticles layer
P3HT: Poly(3-hexylthiophene-2,5-diyl)
PCBM: [6,6]-Phenyl-C_60_ butyric acid methyl ester
PCE: Power conversion efficiency
PL: Photoluminescence
PSCs: Perovskite solar cells
SEM: Scanning electron microscopy
*V*_OC_: Open-circuit voltage
XRD: X-ray diffraction
